# A Comparison of Supervised Classification Methods for the Prediction of Substrate Type Using Multibeam Acoustic and Legacy Grain-Size Data

**DOI:** 10.1371/journal.pone.0093950

**Published:** 2014-04-03

**Authors:** David Stephens, Markus Diesing

**Affiliations:** Centre for Environment, Fisheries and Aquaculture Science, Lowestoft, Suffolk, United Kingdom; Plymouth University, United Kingdom

## Abstract

Detailed seabed substrate maps are increasingly in demand for effective planning and management of marine ecosystems and resources. It has become common to use remotely sensed multibeam echosounder data in the form of bathymetry and acoustic backscatter in conjunction with ground-truth sampling data to inform the mapping of seabed substrates. Whilst, until recently, such data sets have typically been classified by expert interpretation, it is now obvious that more objective, faster and repeatable methods of seabed classification are required. This study compares the performances of a range of supervised classification techniques for predicting substrate type from multibeam echosounder data. The study area is located in the North Sea, off the north-east coast of England. A total of 258 ground-truth samples were classified into four substrate classes. Multibeam bathymetry and backscatter data, and a range of secondary features derived from these datasets were used in this study. Six supervised classification techniques were tested: Classification Trees, Support Vector Machines, k-Nearest Neighbour, Neural Networks, Random Forest and Naive Bayes. Each classifier was trained multiple times using different input features, including i) the two primary features of bathymetry and backscatter, ii) a subset of the features chosen by a feature selection process and iii) all of the input features. The predictive performances of the models were validated using a separate test set of ground-truth samples. The statistical significance of model performances relative to a simple baseline model (Nearest Neighbour predictions on bathymetry and backscatter) were tested to assess the benefits of using more sophisticated approaches. The best performing models were tree based methods and Naive Bayes which achieved accuracies of around 0.8 and kappa coefficients of up to 0.5 on the test set. The models that used all input features didn't generally perform well, highlighting the need for some means of feature selection.

## Introduction

High resolution maps of benthic substrate and habitat are in increasing demand both to underpin environmental and socio-economic impact assessments and to help in the development of effective management measures. The advent of swath acoustic techniques has revolutionised seabed mapping science, as we are now able to map the seabed at high spatial resolution and accuracy. The availability of multibeam echosounder (MBES) data was limited until recently, due to the high costs involved. The situation is about to change however, with some countries (Ireland and Norway) executing dedicated large-scale seabed mapping programmes while, in others, MBES data are routinely collected for specific purposes (e.g. hydrographic charting), but are also made available for seabed mapping. In the United Kingdom, the amount of MBES data has significantly increased in recent years to 200,000 km^2^, approximately 26% of the Exclusive Economic Zone [1], mainly driven by the Civil Hydrography Programme (CHP) run by the Marine and Coastguard Agency (MCA). The improvement in quality of MBES data, as well as the increase in volume of data and the demand for mapping products, has driven the desire for validated, repeatable and applicable approaches to creating maps of the physical properties of the seabed, analogous to the automated classification of satellite imagery in terrestrial remote sensing. Despite this, the science of acoustic seabed classification based on objective, statistical classification procedures is still in its infancy [2].

Traditionally, acoustic data were classified into different acoustic facies by expert interpretation [3], a process that is highly subjective, time-consuming and lacking repeatability. Studies describing automated approaches to seabed classification are still limited and do not extend back in time beyond the last ten years. Such approaches can generally be divided into two groups: unsupervised and supervised classification. Unsupervised classification attempts to find regularities in unclassified data. In remote-sensing applications, an image is classified based on natural groupings of the spectral properties of the pixels. Typical unsupervised procedures are clustering techniques, e.g. k-Means and hierarchical agglomerative clustering. Seabed mapping studies that employed unsupervised classification have been presented by several authors [4]–[8]. One common problem associated with unsupervised classification is the determination of the ‘correct’ or ‘optimum’ number of clusters. A large number of criteria for determining the ‘optimum’ number of clusters exist. For example, Milligan and Cooper [9] tested 30 criteria for their ability to predict the ‘correct’ number of clusters in a data set. Another potential drawback is that the resultant classification rarely shows a 1-to-1 relationship with classes derived from ground-truth data [6], [10]. If the aim is to map substrates or habitats according to a classification scheme, then cross-tabulation and aggregation needs to be carried out so that the classified acoustic data reflect the classes found in the ground-truth data.

Supervised classification techniques are algorithms that ‘learn’ patterns in data to predict an associated discrete class. They are flexible statistical prediction techniques collectively referred to as machine learning techniques. Machine learning is defined as “programming computers to optimise a performance criterion using example data or past experience” [11]. Terrestrial remote sensing has successfully employed these techniques for optical data for several years and they are being used more regularly in seabed mapping using acoustic data. Supervised machine learning techniques that have been trialled include Maximum Likelihood Estimation [12]–[14], k-Nearest Neighbour [15], various decision trees [13], [14], [16]–[19], Random Forest [14], [15], Artificial Neural Networks [20], [21], Support Vector Machines [14] and Bayesian Decision Rules [22]. It is apparent from the above that a large number of supervised classification methods are available for seabed mapping. However, it is often difficult to make an informed decision regarding the most appropriate method for a specific task and it appears that the choice of machine learning techniques is often based on personal preferences.

The aims of this study were to test and compare six of the above-mentioned supervised classification algorithms for their ability to predict substrate type using MBES data and legacy sediment samples. A set of 15 input features (predictor variables) were created from acoustic mosaics of bathymetry and backscatter data. Each classifier was trained three times using different sets of the input features. The classifiers were trained using i) the two primary acoustic features of bathymetry and backscatter, ii) a subset of the features chosen by a feature selection process and iii) all of the input features. The aim of this was to test how much, if any, predictive power is gained from incorporating more secondary features. This resulted in a total of 18 models being trained.

As well as the model performance relative to one another it is of interest to investigate how well models performed relative to a baseline. The aim was to determine whether a significant improvement in predictive performance was achieved by using more complex classification algorithms and incorporating more input features, both of which take computational effort to implement. We selected a simple baseline model which predicts substrate class using the ‘nearest’ (i.e. most similar) training sample neighbour based on bathymetry and backscatter. All models were compared against this baseline prediction to assess the statistical significance of improvements in predictive power. The performance of different classification methods was tested using an independent test set of sediment samples.

## Methods

### Study area

**Figure 1:**
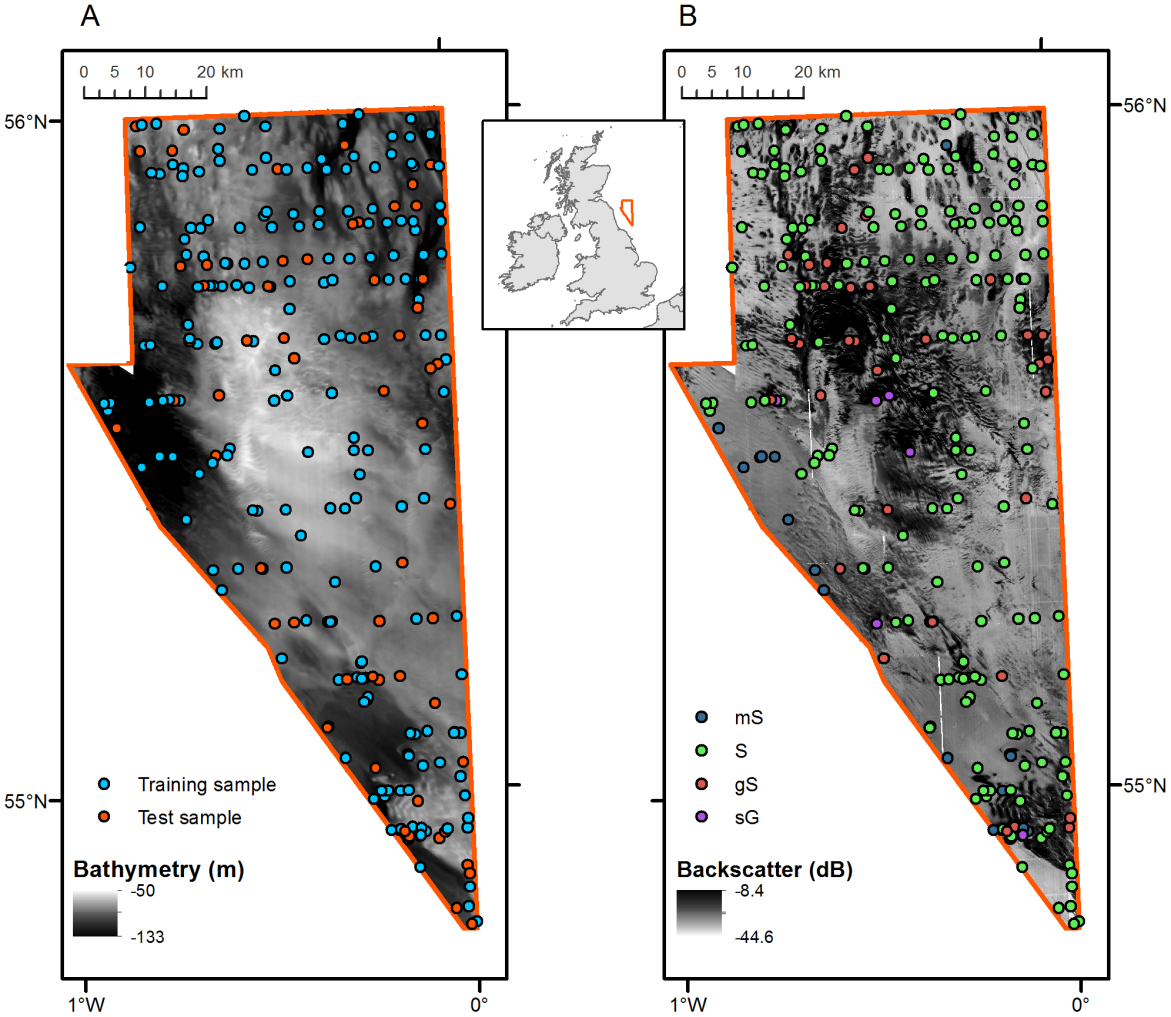
Study area, acoustic data and ground-truth samples. A: Bathymetry data with ground-truth samples overlaid, colours indicating the test and validation sets. B: Backscatter data with ground-truth samples overlaid, colours indicating the substrate class.

The study area is located in the North Sea lying off the north-east coast of England (Figure 1). The water depth of the site ranges from 55 to 100 m and it is dominated by sandy substrate types. The size of the area is approximately 5,272 km^2^.

### Ground-truth data

The ground-truth data are a subset of a legacy dataset of the British Geological Survey (BGS Legacy Particle Size Analysis uncontrolled data export (2011), British Geological Survey, www.bgs.ac.uk), comprising 258 samples (Figure 1). The samples were collected prior to the introduction of the Global Positioning System (GPS) using the Decca Main Chain system and substantial positional errors are to be expected. The samples were collected using a Shipek type sediment grab. The substrate types were classified according to a modified version of the Folk classification [23], based on the relative proportions of the three size fractions Mud (grain diameter d<63 μm), Sand (63 μm<d<2 mm) and Gravel (d>2 mm). Four textural classes were present in the dataset; Muddy Sand (**mS**; <5% gravel and sand∶mud ratio of between 1∶1 and 9∶1), Sand (**S**; <5% gravel and sand∶mud ratio of at least 9∶1), Gravelly Sand (**gS**; 5–30% gravel and sand∶mud ratio of at least 9∶1) and Sandy Gravel (**sG**; 30–80% gravel and sand∶mud ratio of at least 9∶1). The class frequencies were unbalanced, with approximately 80% of the samples being labelled as **S**. Twenty-five percent of the samples were removed from the dataset to use as a test dataset to validate the final models. The sub sampling was done in a randomized stratified way (based on substrate class) so that the test set contained approximately the same class frequencies as the training set (see Table 1).

**Table 1:** Ground-truth data.

Class	Training set	Test set	Total
mS	16	2	18
S	144	50	194
gS	29	10	39
sG	5	2	7
Total	194	64	258

### Acoustic data sets

Primary features. MBES bathymetry and backscatter data were obtained for an area called ‘Bayman's Hole to Dunbar’ collected as part of the United Kingdom's CHP. The data were acquired from 1^st^ May to 17^th^ December 2008 in accordance with IHO S44 ed. 4 Order 1 employing a Kongsberg EM710 system (frequency range 70–100 kHz). The acoustic data can be downloaded from the UK Hydrographic Office INSPIRE Portal and MEDIN Bathymetry Data Archive Centre (http://www.ukho.gov.uk/inspire/Pages/home.aspx). The acoustic data were received in raw (*.all) and generic sensor format (*.gsf). Backscatter processing was carried out using the raw data and software Fledermaus Geocoder Toolbox (FMGT). Generic sensor format files were processed in software Fledermaus yielding a bathymetric surface. Bathymetry and backscatter raster grids were exported and resampled to a uniform grid at 10 m resolution. A Gaussian filter (kernel size 5×5) was applied to both datasets prior to analysis in order to reduce the effect of noise.

As we are using legacy data in this study, we performed a preliminary investigation into the training data to explore how well the acoustic data had been sampled. The ground-truth samples were collected prior to the acoustic survey and therefore not targeted to sample the acoustic data in a representative way. By plotting the density estimations of the raster grids against the sampling data, we were able to visually compare the distributions and assess how representatively the sampling of the acoustic bathymetry and backscatter values were captured in the training data.

#### Derived secondary features

A range of secondary features were calculated from the bathymetry and backscatter datasets. These are intended to contextualise each individual pixel by describing how it is situated in relation to its local neighbourhood. The choice of secondary features was informed by experience gained in previous studies [6], [12], [13], [17], [24], [25].The selection of derived features was also influenced by their expected relationships with seabed substrate classes: Previous trials had indicated that roughness of backscatter aids in separating coarse substrates from finer ones. This could be expected as coarse substrates are not only characterised by high backscatter but also an increased local variability in the backscatter response. Likewise, it could be expected that the bathymetric roughness in such areas is higher than in areas characterised by relatively smooth sandy or muddy seabed. Seabed curvature and the bathymetric position index (BPI) are deemed to be important for sedimentation in terms of exposure to waves and currents. Moran's I is a measure of spatial-correlation. It is related to structure or texture of the seabed. A high Moran index would indicate highly structured bathymetry/backscatter values (i.e. high values are associated with high and low values with low), conversely a lower Moran index indicates a more random spatial distribution of values. It is conceivable that certain types of seabed (such as subaqueous dunes) have a particular signal of spatial structure/texture associated with them and this may be captured by Moran's I. The Sobel filter identifies image gradient (i.e. slope) in a local neighbourhood. It can be used to identify gradient in a particular direction as opposed to maximum rate of change which is directionless. The Sobel filter was calculated for bathymetry to highlight areas of high gradient in both the north-south and east-west directions. A wide array of features was initially created and inspected. However, some features (e.g. rugosity) were subsequently removed as they were strongly correlated with other features. BPIs were calculated over a range of neighbourhood sizes ranging from 100 m to 700 m with a step size of 100 m. The resulting raster layers were closely inspected and BPIs with neighbourhood sizes of 200 m and 600 m were selected due to their ability to highlight certain terrain features, namely subaqueous dunes (200 m) and channels (600 m). These terrain features were found to be widespread and expected to be associated with specific seabed substrates, namely sand on dunes and muddy sand in channels. Thirteen secondary derived features were ultimately selected for further analysis (Table 2). An inspection of the linear correlation between all input features (Table 3) was made to ensure that there were no redundant features being included.

**Table 2:** Secondary acoustic features generated from bathymetry and backscatter.

Derivative	Description	Layer names
Bathymetric position index (BPI)	The vertical difference between a cell and the mean of the local neighbourhood [53]. BPIs were calculated using 200 m and 600 m radii.	BPI_600m; BPI_200m
Roughness	The difference between the minimum and maximum of cell and its 8 neighbours [24]. Roughness was calculated for bathymetry and backscatter.	backscatter_roughness, bathymetry_roughness
Curvature	Curvature describes the rate of change of the slope. [24]. Profile curvature is measured in the parallel to direction of maximum slope. Plan curvature is measured in the perpendicular to direction of maximum slope.	curvature, curvature_planar, curvature_profile
Northness	Equals the cosine of the aspect, which is the direction of the steepest slope measured in clockwise degrees from north.	northness
Eastness	Equals the sine of the aspect.	eastness
Moran's I	Spatial auto-correlation in a 5×5 neighbourhood [54]. Moran's I was calculated for bathymetry and backscatter.	bathymetry_moran, backscatter_moran
Sobel filter	A directional filter that emphasises areas of large spatial frequency (edges) running horizontally (X) or vertically (Y) across the image.	bathy_sobelY, bathy_sobelX

**Table 3:** Correlation matrix of input features.

	f1	f2	f3	f4	f5	f6	f7	f8	f9	f10	f11	f12	f13	f14	f15
f1	1	0.24	0.33	−0.67	−0.26	0.01	0.14	0.03	0.15	−0.14	−0.12	0.03	0.52	0.28	0.29
f2	0.24	1	0.8	−0.21	−0.05	0.02	0.13	0.05	0.01	−0.43	0.4	0.52	0.2	0.15	0.12
f3	0.33	0.8	1	−0.3	−0.11	0.04	0.05	0.06	−0.01	−0.29	0.17	0.3	0.3	0.24	0.17
f4	−0.67	−0.21	−0.3	1	0.14	0.02	−0.05	0.02	−0.07	0.15	0.05	−0.07	−0.23	−0.01	−0.24
f5	−0.26	−0.05	−0.11	0.14	1	−0.31	−0.36	−0.16	−0.13	0.03	−0.06	−0.06	0.1	0.1	0.13
f6	0.01	0.02	0.04	0.02	−0.31	1	−0.03	0.7	0.03	0.02	−0.02	−0.02	−0.01	−0.08	−0.09
f7	0.14	0.13	0.05	−0.05	−0.36	−0.03	1	0.05	0.69	−0.11	0.23	0.21	0.02	−0.02	0.06
f8	0.03	0.05	0.06	0.02	−0.16	0.7	0.05	1	0.09	−0.02	0.04	0.04	0.14	0.05	−0.07
f9	0.15	0.01	−0.01	−0.07	−0.13	0.03	0.69	0.09	1	0.02	0.06	0.02	0.07	0.07	0.02
f10	−0.14	−0.43	−0.29	0.15	0.03	0.02	−0.11	−0.02	0.02	1	−0.27	−0.84	−0.03	−0.02	−0.03
f11	−0.12	0.4	0.17	0.05	−0.06	−0.02	0.23	0.04	0.06	−0.27	1	0.75	0.01	−0.06	−0.02
f12	0.03	0.52	0.3	−0.07	−0.06	−0.02	0.21	0.04	0.02	−0.84	0.75	1	0.03	−0.02	0.01
f13	0.52	0.2	0.3	−0.23	0.1	−0.01	0.02	0.14	0.07	−0.03	0.01	0.03	1	0.62	0.26
f14	0.28	0.15	0.24	−0.01	0.1	−0.08	−0.02	0.05	0.07	−0.02	−0.06	−0.02	0.62	1	−0.07
f15	0.29	0.12	0.17	−0.24	0.13	−0.09	0.06	−0.07	0.02	−0.03	−0.02	0.01	0.26	−0.07	1

f1 = bathymetry, f2 = BPI200, f3 = BPI600, f4 = bathymetry_moran, f5 = bathymetry_roughness, f6 = bathy_sobely, f7 = bathy_sobelx, f8 = Northness, f9 = Eastness, f10 = Curvature_profile, f11 = Curvature_planar, f12 = Curvature, f13 = backscatter, f14 = backscatter_moran, f15 = backscatter_roughness.

### Classification methods

Initially, we carried out a literature review on supervised classifiers employed in seabed mapping [12]–[22]. Of the seven different classes of classifiers used, decision tree learning was by far most popular. With the exception of Maximum Likelihood Estimation, which showed poor performance in previous comparative studies [13], [14], we included all the supervised classifiers applied in the above mentioned publications in the present study. Each classifier was trained three times using different input features. Each model is referred to by its abbreviation (such as CT for classification tree) with an appended number. The number refers to the level of input features used in the model. 1 – indicates only primary features (bathymetry and backscatter) used; 2 – indicates a subset of features chosen by a selection algorithm (described below) and 3 – indicates all features were used. So for example CT1 is a classification tree model trained using primary features. Following is a brief description of each classification method.

### k-Nearest Neighbour (k-NN)

k-NN is one of simplest algorithms tested here and computationally the quickest to implement. The classification is based on a majority vote of the k-nearest neighbours, based on Euclidean distance in feature space, whereby k specifies the number of neighbours to be used. It does not require a training step to be performed but can be tuned to determine the optimum value of k on which to base the classification. The k-NN algorithm is implemented using the class package [26] in R [27]. The training data were scaled so that they had a mean of 0 and standard deviation of 1. This was necessary so that each feature was considered as equally important in the classification.

### Support Vector Machine (SVM)

SVMs aim to define the optimal boundary separating classes in feature space. This decision boundary is called the optimal separation hyperplane. The classification of new data is based on which side of the decision boundary the data point falls. The ‘optimal’ hyperplane is chosen based on the maximum margin principle, by choosing the boundary which maximises the distance between classes. SVMs are able to handle problems where classes are not linearly separable by transforming the data using a kernel function such as the radial basis function (RBF) kernel. The RBF kernel is the most common choice for classification tasks [28], [29] and was used here. While most other algorithms tested here deal with the classification problem using a multiclass approach i.e. considering all classes simultaneously, the SVM classifier used here does not handle multiclass problems directly. It breaks the problem down into a series of binary classification problems using a one-against-one approach so that (*c*-1)/2 binary classifiers are trained (where *c* is number of classes). A majority vote is used to decide the final classification. The SVM was implemented using the e1071 R package [29].

### Classification tree (CT)

The rpart algorithm [30] is a classification tree-based method which makes classification rules by recursively partitioning the data into increasingly homogenous groups. The data is split into smaller and more homogeneous subsets (called nodes) based on thresholds in the predictor features. Homogeneity at each node is measured using the Gini impurity criterion [30]. At each consecutive split, the threshold chosen is based on the split which most reduces overall node impurity. To avoid over-fitting, an internal cross-validation was performed to remove splits which increase cross-validated error, referred to as pruning.

### Random Forest (RF)

The RF algorithm [31] is an ensemble technique which aggregates the results of many randomly constructed classification trees. The trees differ from those produced by rpart because they are not subsequently pruned. Two components of randomness are introduced into the construction of the individual trees. Firstly, each tree is constructed using a random bootstrapped sample of the training data. Secondly, rather than testing all features for the best split, a random subset of variables is tested at each split in each tree. The idea behind introducing the randomness into the construction of the trees and averaging the result over many trees is that the final outcome will be less subject to any random fluctuations in the training dataset and will have an increased capacity for generalising patterns. The prediction is made for unobserved data by taking a majority vote of the individual trees. The samples not part of the bootstrapped sample for each tree, referred to as ‘out-of-bag’ (OOB) samples, are used to create a cross-validated prediction error for the forest. Also, as part of the construction of the random forest, the OOB samples are used to formulate a measure of feature importance. This is done by randomly shuffling the values of each input feature in turn and observing how much the prediction error of the OOB samples increases. The randomForest package in R was used for the implementation [32].

### Artificial Neural Networks (NN)

A single hidden layer neural network classifier was also trained. The construction of this type of model is referred to as network because it can be viewed as three layers of connected nodes. The input layer with a node representing each input feature. The output layer contains a node for each class to be predicted. In between input and output layers is a ‘hidden’ layer of nodes. The number of nodes in the hidden layer is decided during the tuning phase. There are connections between every input node and each node in the hidden layer and subsequent connections between every node in the hidden layer and each of the output nodes. The model is parameterised by weights assigned to each connection. These weights are ‘learnt’ during the training phase. The input to each node of the hidden layer is the sum of weighted values from the input layer (plus a constant bias). This value is then fed into the ‘activation’ function (logistic/sigmoid function). Outputs of the network are interpreted as class probabilities and sum to 1. The training data were scaled so that they had a standard deviation of 1 and mean of 0; this is so they are considered equally in the training process. The final outcome is somewhat dependent on the starting weights, which are chosen at random, and it is recommended that predictions are made using the aggregated results of many trained networks [33]. The outputs of 100 networks were aggregated and used for the final prediction; this choice was based on what was both computationally reasonable and most likely to achieve a stable estimate. The nnet package in R is used for applying the neural network [26].

### Naive Bayes (NB)

The NB classifier calculates class probabilities based on Bayes' rule [34]. It assumes that each input feature is independent and that the probability distribution of each class for each feature is Gaussian and so is the only parametric technique tested here. It does not require tuning or training, so is computationally easy to implement. NB is implemented in the e1071 package in R.

### Feature Selection

In order to identify and remove irrelevant features from the 15 inputs a feature selection algorithm was used. The Boruta algorithm [35] is a wrapper function based on the RF classifier. Wrapper functions [36] identify relevant features by performing multiple runs of the classifier testing the performance of different subsets of input features. The RF algorithm is a suitable choice for this process as it can be used without extensive parameter tuning and returns an estimate of feature importance (Z score). For example, it is possible that a random noise feature could by chance explain some of the variability in the target variable and therefore obtain a positive Z score by a single run of the random forest classifier. To test whether features are significantly important, the Boruta algorithm incorporates random noise features into the classification. The importance scores of the original features are tested against these random features and only features with a significantly higher mean Z score are retained as being relevant.

### Parameter tuning and cross validation

It is an important step to tune the parameters of the models to the training data. The aim is to find a balance between building a model that can classify the training data effectively without over-fitting to the random fluctuations in the training data. Some models are more sensitive than others to the parameters chosen. The RF classifier is usually insensitive to the parameters used (hence its use in the feature selection algorithm), although it can still benefit from some fine tuning. On the other hand, it is not recommended to use SVM without tuning of the input parameters [28].

Model tuning was carried out using a grid search approach in the R package e1071. This involved providing a range of values for each parameter to be tested. A leave-one-out cross validation (LOOcv) was performed on every combination of parameters in the ranges specified. The model parameters that obtained the lowest cross-validated error score were retained for the final predictions. Although this is computationally arduous, cross-validation is still the preferred method of finding optimal parameters [28], [33]. The ranges over which to perform tuning were decided by values recommended in the literature and general ‘rules of thumb’ (see Table 4).

**Table 4:** Model Parameters.

Model	Parameters	Parameter Description	Tuning Range
k-NN	k	The number of neighbours considered in the classification	1∶20
SVM	*C*	The cost parameter determining how much data is included in creating the decision boundary, a small value will consider more observations	2^-5∶15^
	γ	The kernel smoothing parameter which defines the shape and complexity of the resulting decision boundary	2^−15∶5^
RF	nodesize	The minimum number of cases allowed in each of the terminal nodes of each tree	1∶10
	mtry	The number of features tested at each split	2∶15
CT	cp	The *complexity parameter*, it specifies the minimum amount of improvement that must be made in order for a split to take place	2^−10:−1^
	minsplit	Nodes of this size or smaller are no longer split	1∶10
ANN	size	The number of units in the hidden layer	2^1∶6^
	decay	The *weight decay* parameter is used in the training to avoid overfitting	10^−5:−1^

The error criterion being tested during the tuning process was the balanced error rate (BER), which is the average of the proportion of wrong classifications in each class. This was chosen in preference to overall classification error due to the unbalanced class frequencies in the training data [28]. Using BER effectively gives rarer classes a higher weighting so that the model is penalised more heavily for misclassifying a rare class.

### Model Validation

After the grid search model tuning had selected the optimal model parameters, predictions were made on the test set using the chosen models from the tuning process. BER and the Kappa coefficient of agreement [37] were calculated to compare the performance of the models. Kappa is used to compare the performance of classifiers as it provides a more robust measure of agreement than accuracy, because it takes into account the expected agreement by random chance. Classification accuracy (proportion of samples that were correctly classified) was also calculated. If the training and tuning of models has been performed effectively, the error of the test set should reflect the error indicated by LOOcv. This would imply that the model has a capacity to generalise patterns and has not been over-fitted to the training data.

Each model was tested against the baseline model (*1-NN*) to determine the statistical significance of differences in the error measures. Our baseline represents a very simple model and, by using more ‘sophisticated’ classifiers and incorporating more input features, we would expect an increase in predictive performance. Thus we wanted to test whether the error was significantly larger and we used a one-sided test of significance. Monte Carlo type re-sampling illustrated by [38] was used to generate p-values. The test involves performing a large number of permutations (random shuffling) of the class labels in the test set. To calculate the significance of the difference between kappa statistics the following steps were performed: i) For *n* permutations of the test set, kappa statistics for both classifiers were recalculated on the permuted test set and the difference between these two kappa values was calculated. ii) If the difference exceeded the original difference, a counter (*c*) was incremented by 1. iii) The difference in kappa statistics is considered significant (at the 5% level) if the proportion of differences exceeding the original difference (plus 1) is less than 0.05: (*c*+1)/(*n*+1)≤0.05. The same procedure was used to test the significance of the BER. Approximately 1000 permutations are adequate for significance testing at the 5% level [38], [39].

## Results

### Data exploration

**Figure 2:**
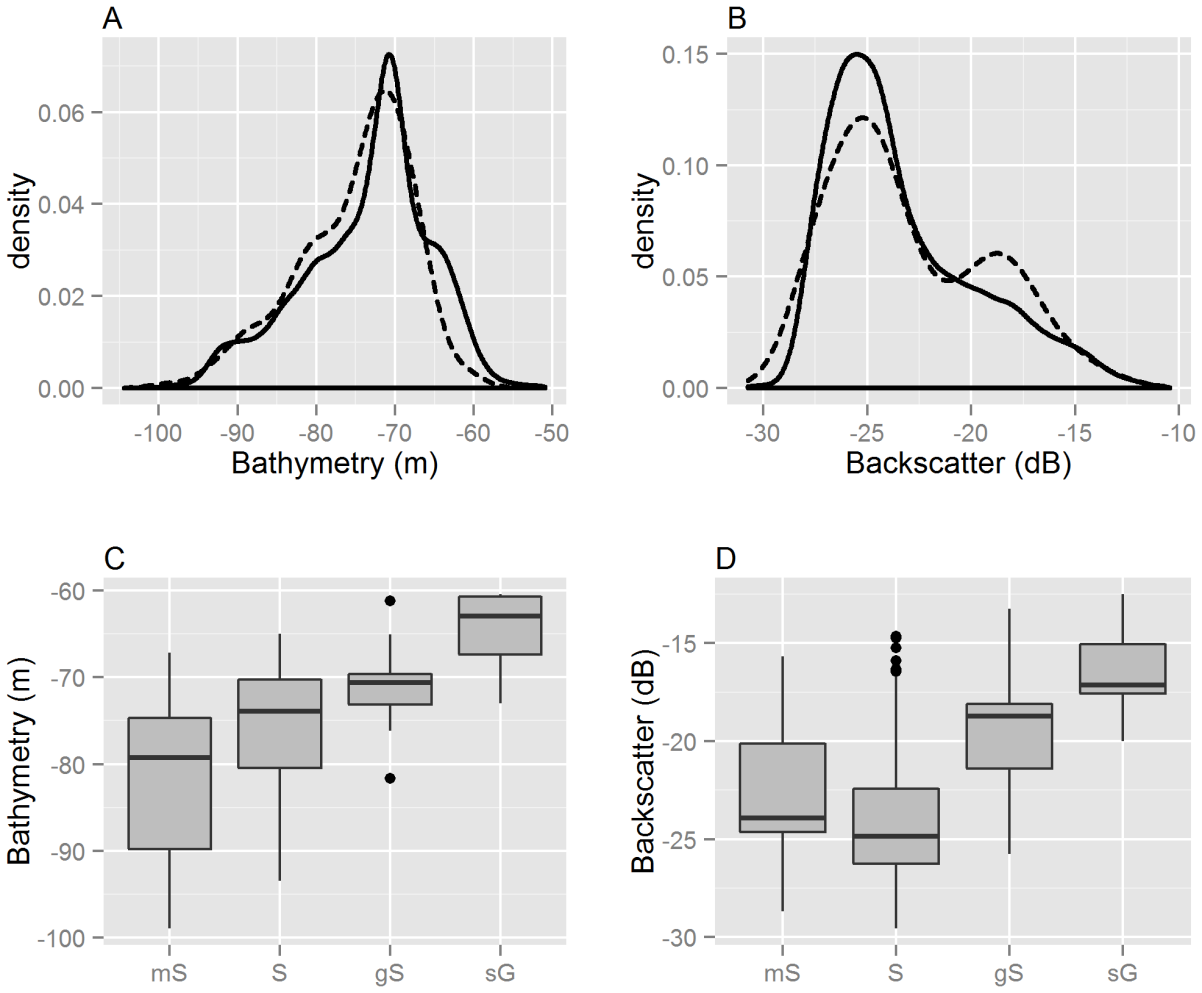
Exploration of training data. A: Comparing the distributions of bathymetry values between training samples (dashed) and raster grid (solid). B: Comparing the distributions of backscatter values between training samples (dashed) and raster grid (solid). C: Comparing the distribution of bathymetry values between substrate classes. D: Comparing the distribution of backscatter values between substrate classes.

Visual inspection of the training samples showed that the distribution of the sampling seemed to approximate the distribution of bathymetry values from the raster grid, although there was a slight under representation of shallower areas>−65 m (Figure 2a). The backscatter data revealed an over sampling of values between −15 and −20 dB and under sampling around −25 dB (Figure 2b). This visual comparison suggested that the sampling appeared to reflect the underlying distribution of the primary acoustic features fairly well.

Further exploration of the substrate types of training data showed that, in general, coarser sediments were associated with higher backscatter and shallower water depths (Figure 2 c and d). However, this generalisation doesn't apply to the **mS** class where the mean backscatter values were slightly higher than that of **S**. The **mS** class had the largest variability in both bathymetry and backscatter values.

### Feature Selection

The Boruta algorithm performed 230 runs of the RF classifier to identify relevant features. From the initial fifteen features, including bathymetry and backscatter, nine were retained as being considered to be significantly important by their mean Z-score (Table 5). The relative importance indicated by the algorithm shows that backscatter is by far the most important feature. Bathymetry and Moran's I of backscatter are also indicated as being important. Features that were not considered significant and dropped included BPI's, eastness and roughness.

**Table 5:** Output from Boruta feature selection algorithm.

Feature	Z-score
backscatter	**34.13**
bathymetry	**15.34**
backscatter_moran	**12.31**
curvature	**12.01**
curvature_planar	**10.79**
bathymetry_moran	**8.67**
curvature_profile	**8.21**
backscatter_roughness	**7.74**
northness	**5.77**
BPI_200m	4.97
BPI_600m	4.93
bathy_sobelY	3.25
bathy_sobelX	1.45
eastness	1.11
bathymetry_roughness	0.79

Scores that were significantly higher (p<0.001) than scores of random features are indicated in bold.

### Model performance

**Figure 3:**
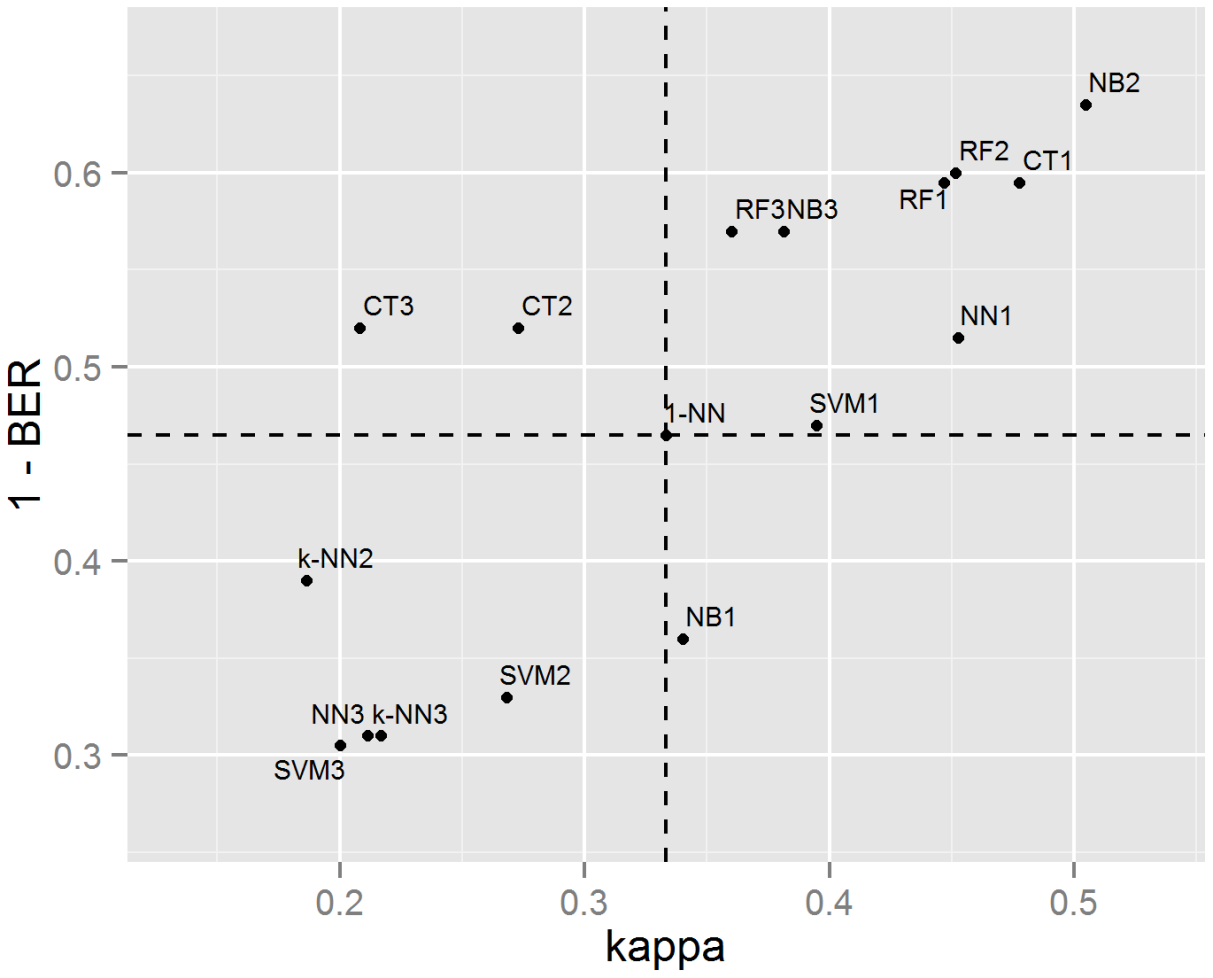
Comparing model performance on the test data. The dashed lines represent the performance of the baseline model. The best performing models are to the top-right.

A total of 18 models were trained and their performances measured using the test data set (Table 6 and Figure 3). Predictions for the test set were also made using *1-NN* baseline model. The results show the *1-NN* model scored a BER of 0.54; it had an accuracy score of 0.77 and a kappa coefficient of 0.33. In total eight models (NB2, RF2, CT1, RF1, NB3, CT3, NN1 and SVM1) performed better than the baseline for all three performance measures.

**Table 6:** Model Performance Comparison.

Model	BER	Accuracy	Kappa
NB2	**0.37**	0.80	0.50
RF2	**0.40**	0.81	0.45
RF1	**0.41**	0.80	0.45
CT1	**0.41**	0.80	**0.48**
RF3	**0.43**	0.78	0.36
NB3	0.43	0.78	0.38
CT3	0.48	0.69	0.21
CT2	0.48	0.69	0.27
NN1	**0.49**	0.80	**0.45**
SVM1	0.53	0.78	0.39
*1-NN*	0.54	0.77	0.33
k-NN2	0.61	0.72	0.19
NB1	0.64	0.75	0.34
SVM2	0.67	0.78	0.27
NN3	0.69	0.78	0.21
k-NN3	0.69	0.78	0.22
SVM3	0.70	0.77	0.20
NN2	0.77	0.73	−0.07

BER, Kappa coefficients and Accuracy statistics calculated on the test set. Values indicated as being significantly better than the baseline (p≤0.05) are indicated in bold. Model numbers indicate the input features used; 1 indicates primary features; 2 indicates subset of features chosen by Boruta; 3 indicates all input features were used. *K*-NN1 is not included as the LOOcv indicated that 1 nearest neighbour gave the best performance making it the same as the baseline *1-NN* model. (NB  =  Naive Bayes; RF  =  Random Forest; CT  =  Classification Tree; NN  =  Neural Network; SVM  =  Support Vector Machine; k-NN =  k-nearest neighbour).

The NB2 model was the best performing model in terms of BER followed by the RF2 model; the CT1 and RF1 models were in joint third place (Table 7 shows error matrices for these models). According to the kappa statistic, the NB2 model was also indicated as being the best performer. This was followed by the CT1 and then three models sharing the same kappa score (RF2, RF1 and NN1). RF2 had the highest accuracy score followed by NB2, CT1 and RF1 sharing the same accuracy scores.

**Table 7:** Error matrices for the three best performing models.

CT1		mS	S	gS	sG	Error
	mS	2	0	0	0	0.00
	S	1	44	3	2	0.12
	gS	1	3	5	1	0.50
	sG	0	1	1	0	1.00

RF2		mS	S	gS	sG	Error
	mS	2	0	0	0	0.00
	S	1	45	4	0	0.10
	gS	0	5	5	0	0.50
	sG	0	2	0	0	1.00

NB2		mS	S	gS	sG	Error
	mS	2	0	0	0	0.00
	S	1	42	7	0	0.16
	gS	0	3	7	0	0.30
	sG	0	0	2	0	1.00

Rows show the predicted class frequencies and columns show the observed frequencies.

Of the eight models outperforming the baseline in all three measures, four were models using only primary features (RF1, CT1, NN1 and SVM1), two models used the subset of features (NB2 and RF2) and two were using all features (RF3 and CT3). Three of the eight were RF models, two were NB models and there was one each of the CT, SVM and NN models. The k-NN model was the only method that did not have any of its models outperforming the baseline. RF was the only method which all three models outperformed the baseline in all three measures. RF and NB were the only methods that using all features outperformed *1-NN*.

Conversely, of the methods performing worse than the baseline according to kappa and BER, three were using all features and three were using the subset of features, indicating that performance of these may have suffered due to the presence of irrelevant features.

The significance testing of the BER and kappa statistics indicate there were a number of models performing significantly better (at the 5% level) than the *1-NN* model. Six models performed significantly better in terms of the BER statistic and two models had p values for the kappa statistic which indicated significance. Interestingly, the highest scoring kappa value of the NB2 model was not considered significant, while the lower scoring NN2 model was indicated as significant. Equally for the BER statistic, the NN1 model was indicated as being significant but the lower BER scores of the NB3 and CT2 models were not.

**Figure 4:**
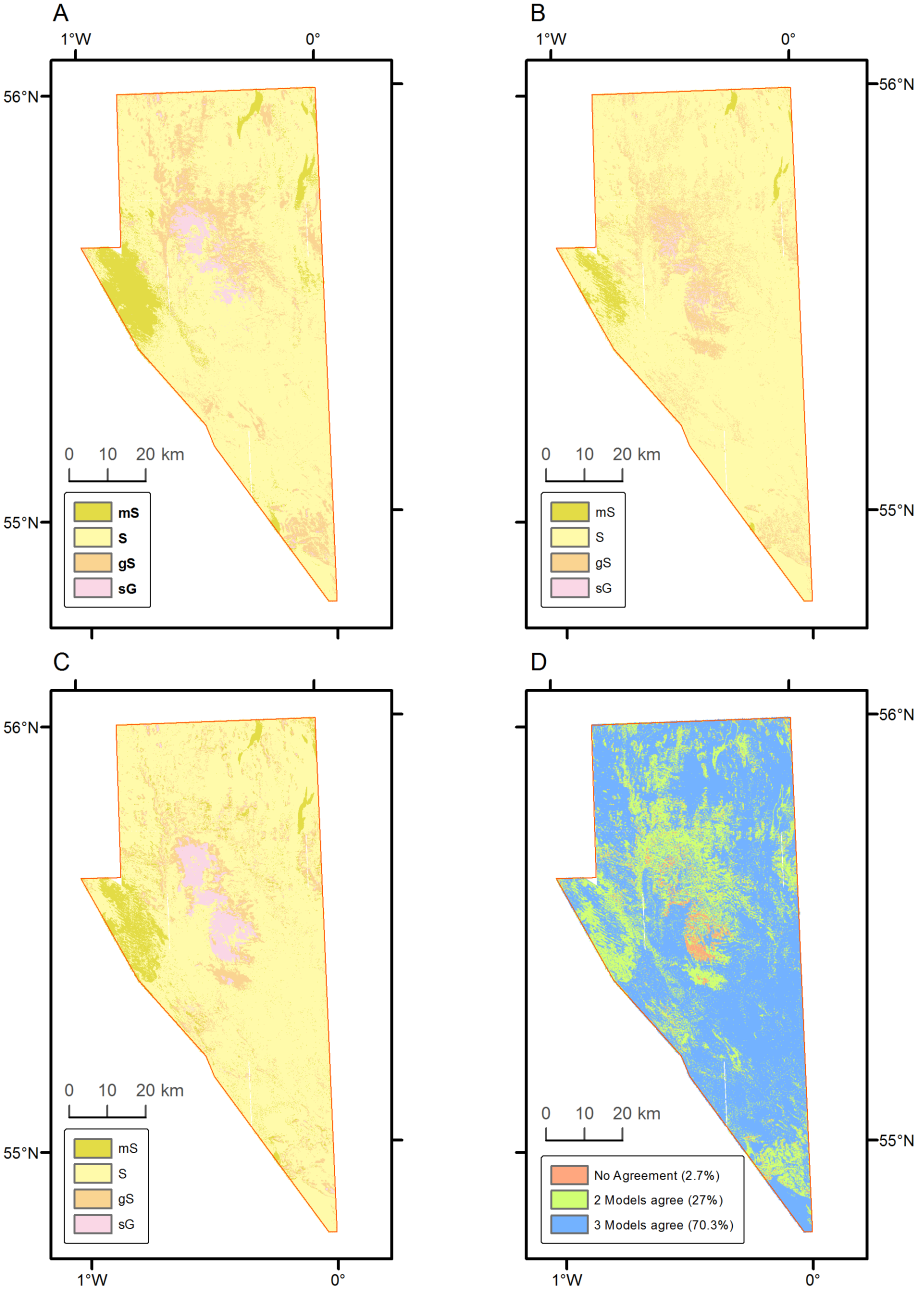
Output predictions from top three models and agreement between them. A: NB2, B: RF2, C∶CT1, D: Agreement.

Predictions of the top three performing models NB2, RF2 and CT1 were output to visualise the differences in model predictions in terms of variability in the spatial extents of different classes and to assess agreement between them. All three models predict the same class for 70% of the area, at least two of the models agree on another 27% of the area (Figure 4). In the remaining 2.7% of area there was disagreement between all three models. There are some clear differences in the area predicted for each class between the models. CT1 for example indicates a larger proportion of **sG** than the other models, with RF2 having very little of the area being predicted as **sG.** Likewise, RF2 seems to predict lesser extent for the **mS** class. The **mS** and **sG** classes represent the extremes of the ground-truth classes, being the finest and coarsest classes, so this suggests that the RF2 model is more ‘conservative’, its estimates being more likely to predict the moderate and more common classes. This is likely a result of the ensemble nature of the RF2 model.

## Discussion

In this study we compared supervised classification methods for the prediction of substrate type using MBES and grain-size data. We compared the performance of six machine learning techniques, as well as the effect of the selection of input features on model performance. The algorithms tested here were chosen based on good performance as indicated in previous studies.

Only two studies have systematically compared supervised classification techniques in the context of classifying MBES data. Ierodiaconou et al. [13] compared two decision tree techniques, QUEST (Quick, Unbiased, Efficient Statistical Tree) and CRUISE (Classification Rule with Unbiased Interaction Selection and Estimation), with Maximum Likelihood Estimation (MLE). The authors evaluated the performances of the three classifiers in predicting benthic biological communities. They found that QUEST was outperforming the other classifiers with the highest thematic accuracy and kappa statistic. QUEST was also providing consistently better results across six of the eight habitat categories. Che Hasan et al. [14] compared four different supervised classification methods: MLE, QUEST, RF and SVM were evaluated to classify angular backscatter response into habitat classes using training data acquired from underwater video observations. Results for substrate classifications indicated that RF and SVM produced the highest accuracies, followed by QUEST and MLE, respectively.

SVMs have been used in various studies and have often performed well [14], [40]. However, Li et al. [41] reported them as not performing as well as other methods, and that was the case in our study, with none of the SVM models performing significantly better than the baseline. One reason for the poor performance of SVMs might be due to insufficient tuning of the free parameters.

RF has been used previously for mapping benthic substrates and habitats. Generally, the good performance by the RF classifier indicated in this study is in keeping with previous studies that have shown similar good performances [14], [15], [41]. The RF algorithm is not severely affected by the inclusion of many input features, even if some are noisy or redundant [41]. This was also apparent in this study, as the RF3 model was the only model using all input features that performed significantly better than the baseline. Classification trees also performed well in this study, which is consistent with previously reported results [13], [14], [41].

NB is the only parametric model used in this study and it achieved the highest accuracy scores, performing significantly better than the baseline. In a similar study, Lüdtke et al. [40] reported the NB classifier not performing as well as other tested methods including SVM, CT and k-NN. Likewise, Che Hasan et al. [14] tested a parametric MLE against non-parametric models RF, SVM and classification trees, with the non-parametric techniques performing better. However, despite the ‘naive’ assumptions made by the model, it has been shown that, given real world applications, this simple classifier often performs well [33]; a finding that was confirmed by this study. John and Langley [34] recommended that a kernel density estimation should be used to approximate the probability distributions of features, rather than assuming the distributions are Gaussian, as was the case here. This could further improve results of the NB classifier in this study.

The feature selection algorithm is based on the RF classifier. This may give the RF2 model an unfair advantage, as it is using features that were selected based on its own feature importance scores. Generally, there are two possible options for feature selection. The first option is to use a technique that does not rely on any particular classifier but removes irrelevant/redundant features in a pre-processing step; this is referred to as a filter approach. Such an approach may be more valid for model comparison purposes as it is independent of information from the classifiers used [42] However, one significant problem with filter approaches is that seemingly irrelevant features are disregarded, when in fact they might be relevant when used in combination with other features. Methods that score variables individually and independently cannot determine which combination of features gives the best performance [43]. The crucial aspect about using RF as a basis for feature selection is that feature interactions are taken into account. Another, perhaps preferable option would be to apply a feature selection wrapper for each specific classifier [28], [44]. This would involve using each classifier as a ‘black box’ and assess performance on subsets of features. The problem with this approach is basically computational. The classifier used in this process should be simple, efficient and crucially, be usable without user defined parameters [43]. Other classifiers tested here, particularly NNs and SVMs generally require parameter tuning in order to perform effectively. This would mean using them for a feature selection process of tuning parameters over every subset of features, which becomes unfeasible with anything more than a few features. The Boruta feature selection algorithm was used here as a general way to reduce the number of features to a set that is likely to be relevant. Most of the models using all features performed poorly, highlighting the importance of some form of feature selection. The fact that the best model was NB2, performing better than RF2, suggests that the features selected by the Boruta algorithm were in some way relevant to other methods, however, further development should incorporate model specific feature selection procedures.

Bathymetry and backscatter were, perhaps unsurprisingly, identified as the most important features. Previous studies have reported local variability in backscatter as being important for predicting substrate classes [15], [45]. The inclusion of benthic morphology features was also identified as important for substrate predictions [18], [20], [41]. Our results indicate that curvature and backscatter roughness are important features (Table 5), in line with previous studies. Against expectations, bathymetry roughness was not selected by the Boruta algorithm. This might be related to the relatively coarse cell size of 10 m×10 m (compared to the area of seabed sampled by the grab), which might have reduced small-scale variability in bathymetry by averaging. Local Moran's I spatial autocorrelation measure of backscatter and bathymetry was considered among the most important features, higher than roughness of bathymetry or backscatter, suggesting that the spatial configuration of the variability is at least as important as the variability itself and is related to textual analysis of acoustic data [4]. This is a feature that, to our knowledge, has not been included in other studies of substrate mapping but appears to be promising.

Further secondary features derived from angular range analysis [46] or multi-scale terrain analysis [24] might also be included. Promising results have been reported by Che Hasan et al. [14], [19], who combined angular range analysis and image segmentation with supervised classification. We felt however that we had to limit the number and types of input features to keep the study manageable. A decision was made to limit the secondary features to those that are easily derived with standard GIS software and have been frequently applied in other studies in order to make this study relevant for a wide audience.

This study focused solely on the prediction performance of the classifiers. Not taken into account was the time taken in the tuning and training stages for the classifiers. There was, however, a large variation in the amount of time taken during those stages for the different models. In general terms, SVMs and NNs were the most costly in terms of time (the tuning phase took a number of hours for each model). NB and k-NN required no tuning step and so were computationally cheap to implement (a matter of seconds). The tree based methods, including RF, fell somewhere between these extremes. These observations are generally confirmed by values reported in [40]. It is important to take computational time into account. This is of particular significance here because the NB model not only was the best model in terms of predictive power, but was also among the fastest to implement, as no tuning or training step was required. Therefore, based on classification accuracy and computational time, our study suggests that NB, RF and CT are the best performing methods.

Alternatives to classification accuracy were also used to assess the performance of the models. If we consider a hypothetical example; an overall classification error (1 - accuracy) of 0.22 would be achieved if every sample in the test was classified as **S**. The BER of this scenario would be 0.75 (3/4). The best performing model in this study achieved an overall classification error of 0.20, approximately a 9% relative improvement on the above scenario. However the best performing model obtained a BER of 0.37, a relative improvement of 51% on the above. This highlights the value of using an alternative to classification error in assessing performance when class frequencies are very uneven. Using overall classification accuracy in this case added little to the understanding of which classifiers performed best at predicting rarer classes, as there was little variability in the overall accuracy scores between models. The BER and kappa statistics better reflected the variability in model performance. Models such as SVM2, NN3 and k-NN3 obtained relatively high overall accuracy scores above the baseline model and very close to those of the best performing models. Nevertheless, based on the kappa statistic and BER, they were seen as the poorest performing models.

It is encouraging to observe the consensus between the top three models. When the predictions were output across the entire area, at least two out of three models agreed on the classification of 97.3% of the study area. The area where all three models disagree is spatially isolated and generally aggregated within a single patch. This discrepancy was predicted as **S** by the NB2 model, **gS** by the RF2 model and **sG** by the CT1 model. The backscatter values in this area are in the region of −16 to −19 db and the bathymetry values are in the vicinity of −63m. As was suggested previously, figure 2a indicates that the shallower areas>−65m are not adequately captured in the training data and this could be a reason for the confusion between the different model estimates.

Some general limitations of the data should be considered as we were using a legacy dataset. Firstly, the input features are aggregated to a particular spatial resolution. These are dictated by technical limitations of the sampling equipment (multibeam sampling density) and more significantly the computing power available for processing the data. The bathymetry and backscatter grids were aggregated to a resolution of 10 m. It is realistic to expect considerable variability of sediment type within an area of 100 m^2^ of sea bed, but the sediment observations were not sampled at the same scale. A sediment grab samples perhaps ≈0.04 m^2^ of sea bed; this is the support size of the data and it is different from the support size of the estimates we are making. Averaging quantities over a large area has the effect of reducing their variance and making the distributions more normal [47]. The implications are that it is unrealistic to expect to account for all the variability in our observations when the predictor variables have a coarser resolution (larger support size) than the sampled data. Future studies should investigate the effect of varying the cell sizes of the acoustic data on model performance.

Secondly, the seabed is subject to hydrodynamic processes which will cause temporal variability of substrate composition at a particular location. An unknown amount of error can be attributed to the fact that the observations are of varying ages and, crucially, the fact that the acoustic data were not collected at the same time as the sediment observations (possibly decades afterwards). While the models have been trained to fairly effectively predict what is observed in the data, does this reflect what is currently the reality on the ground? A follow up survey to validate the model predictions would be the best way to determine this. On the other hand, water depths range between 55 m and 100 m, making the site unsusceptible to wave agitation except during extreme and rare events. Additionally, tidally induced bottom shear stresses are relatively weak [48]. Consequently, remobilisation of seabed sediments is likely to occur only episodically. There is also evidence, from a site in the North Sea, that sediment distribution patterns with high grain-size contrast similar to those in this study area might remain stable over decades [49].

Lastly, and perhaps the most significant cause of error to consider within this study, is the positional accuracy of the ground-truth samples, which were collected before the introduction of GPS and relied on the DECCA navigation system to determine position. The DECCA system was based on triangulation from land based radio transmitters. While it is difficult to estimate the magnitude of the positional error, as the accuracy of the DECCA system varies not only with distance from the land-based transmitting station but is also dependant on the season and the time of the day [50], repeatable accuracies are assumed to be better than 400 m with 95% confidence [51]. With this positional accuracy indicated and the raster resolution of the acoustic data being only 10 m, it is likely that incorrect raster values will have been extracted for the ground-truth samples. In areas of seabed that are homogenous on length scales similar to the accuracy of the data this may not have introduced too much error. In areas that have a higher variability at smaller spatial scales, this will have been more of an issue. One way to incorporate the known spatial inaccuracies of up to 400 m may be to use a simulation approach whereby many models were fitted to training data that used ‘jittered’ sample positions (i.e. samples are randomly relocated based on a bivariate probability function around the original location). Alternatively, a buffer could be applied to each sample thereby exporting a range of possible values for each sample. This would produce a considerably larger training set and tuning/training times of the various algorithms would be more of an issue. How best to validate this approach would also require consideration. Dealing with these issues of positional inaccuracy including the quantitative identification of outliers in the sampling data is an important topic but beyond the scope of this study.

A further limitation is the unavoidably restricted range of substrate types encountered in the study site. We attempted to select a site with reasonable variability in seabed substrates, their associated backscatter response and water depths. The substrates ranged from muddy sand to sandy gravel. Finer sediments such as mud and sandy mud, mixed sediments, exposed bedrock, biogenic reef, and macrophyte-dominated sediment etc. were not encountered. The insights gained from this study might therefore be limited to the observed substrate types. It is obviously not possible to address all potential issues regarding classification approaches, accuracy of input data, effect of support size, range of substrate types and many more in one single study. Rather, we see this work as a contribution towards building a body of evidence that will eventually guide researchers in selecting appropriate classification methods for specific mapping tasks. With only two comparable studies published [13], [14], we are still at the beginning of this process and would urge other researchers to carry out similar comparative studies. We would also strongly suggest that researchers make their input data sets, i.e. at least seabed substrate class or composition and associated extracted feature values for all sample stations, freely available. This will facilitate seabed mapping research and eventually might give us the opportunity to carry out meta-analyses, once the body of evidence has gained a critical mass. Respective data from this study will be made accessible as supplementary material.

Despite all these limitations, the best performing models (NB2, RF2 and CT1) yielded satisfactory results with overall classification accuracies around 0.8, BERs of 0.37 to 0.41 and kappa statistics of up to 0.5. This indicates that legacy grain-size data can be successfully employed to ground-truth MBES data when spatially predicting seabed substrate distributions. Future work should be directed towards a better quantification of positional errors introduced by the use of the DECCA navigation system, the effect of positional errors on classification accuracy and the applicability of a multi-model ensemble approach to seabed substrate predictions. The results of several models could be combined using a simple voting procedure to determine class allocation. Although this might not necessarily lead to improved overall classification accuracy when compared with the best performing model, this approach has advantages, as it is difficult to specify the most appropriate classifier in advance. In addition, the ensemble approach yields class-allocation uncertainty information [52].

## Conclusions

The aims of this study were to test and compare six supervised classification algorithms for their ability to predict substrate type using MBES and ground-truth data. While the ideal solution would be to undertake a dedicated survey, collecting acoustic data first and basing a subsequent ground-truth survey on these data to ensure the acoustic areas are representatively sampled, this is not always achievable and that is why we looked at the potential for using legacy grain-size data with existing MBES data. We have shown that satisfactory results can be obtained from using legacy data.

Comparing the models tested here against a simple baseline model showed that incorporating secondary derived features increased predictive power, although, while the best performing models included features derived from bathymetry and backscatter, models that used all the input features did not perform well, indicating the need for some form of feature selection to remove irrelevant features. The best performing model indicated in this study (NB2) is theoretically simple and computationally cheap to implement. This suggests that simpler, lightweight models should be tested first and, if they prove to be of sufficient accuracy, then using more sophisticated techniques which take more time to implement will not be necessary. The unbalanced class frequencies in the ground-truth data have implications for the accuracy assessment of the models. Use of accuracy or classification error added little to our understanding of which models performed well and alternatives such as BER or kappa coefficient were found to be preferable.

## Supporting Information

Table S1 **Training and test data.**
(CSV)

## References

[1] Wynn RB, Bett BJ, Evans AJ, Griffiths G, Huvenne VAI, et al. (2012) Investigating the feasibility of utilizing AUV and Glider technology for mapping and monitoring of the UK MPA network. Southampton: National Oceanography Centre. 244 p.

[2] Anderson JT, Van Holliday D, Kloser R, Reid DG, Simard Y (2008) Acoustic seabed classification: current practice and future directions. ICES Journal of Marine Science 65: 1004–1011.

[3] Brown CJ, Smith SJ, Lawton P, Anderson JT (2011) Benthic habitat mapping: A review of progress towards improved understanding of the spatial ecology of the seafloor using acoustic techniques. Estuarine, Coastal and Shelf Science 92: 502–520.

[4] Blondel P, Gomez Sichi O (2009) Textural analyses of multibeam sonar imagery from Stanton Banks, Northern Ireland continental shelf. Applied Acoustics 70: 1288–1297.

[5] Brown CJ, Collier JS (2008) Mapping benthic habitat in regions of gradational substrata: An automated approach utilising geophysical, geological, and biological relationships. Estuarine Coastal and Shelf Science 78: 203–214.

[6] Brown CJ, Sameoto JA, Smith SJ (2012) Multiple methods, maps, and management applications: Purpose made seafloor maps in support of ocean management. Journal of Sea Research 72: 1–13.

[7] Lathrop RG, Cole M, Senyk N, Butman B (2006) Seafloor habitat mapping of the New York Bight incorporating sidescan sonar data. Estuarine, Coastal and Shelf Science 68: 221–230.

[8] McGonigle C, Brown C, Quinn R, Grabowski J (2009) Evaluation of image-based multibeam sonar backscatter classification for benthic habitat discrimination and mapping at Stanton Banks, UK. Estuarine Coastal and Shelf Science 81: 423–437.

[9] Milligan GW, Cooper MC (1985) An examination of procedures for determining the number of clusters in a data set. Psychometrika 50: 159–179.

[10] Lathrop RG, Cole M, Senyk N, Butman B (2006) Seafloor habitat mapping of the New York Bight incorporating sidescan sonar data. Estuarine Coastal and Shelf Science 68: 221–230.

[11] Alpaydin E (2010) Introduction to Machine Learning. Cambridge, MA: MIT Press. 537 p.

[12] Buhl-Mortensen P, Dolan M, Buhl-Mortensen L (2009) Prediction of benthic biotopes on a Norwegian offshore bank using a combination of multivariate analysis and GIS classification. Ices Journal of Marine Science 66: 2026–2032.

[13] Ierodiaconou D, Monk J, Rattray A, Laurenson L, Versace VL (2011) Comparison of automated classification techniques for predicting benthic biological communities using hydroacoustics and video observations. Continental Shelf Research 31: S28–S38.

[14] Che Hasan R, Ierodiaconou D, Monk J (2012) Evaluation of Four Supervised Learning Methods for Benthic Habitat Mapping Using Backscatter from Multi-Beam Sonar. Remote Sensing 4: 3427–3443.

[15] Lucieer V, Hill NA, Barrett NS, Nichol S (2013) Do marine substrates ‘look’ and ‘sound’ the same? Supervised classification of multibeam acoustic data using autonomous underwater vehicle images. Estuarine, Coastal and Shelf Science 117: 94–106.

[16] Dartnell P, Gardner JV (2004) Predicting seafloor facies from multibeam bathymetry and backscatter data. Photogrammetric Engineering and Remote Sensing 70: 1081–1091.

[17] Rattray A, Ierodiaconou D, Laurenson L, Burq S, Reston M (2009) Hydro-acoustic remote sensing of benthic biological communities on the shallow South East Australian continental shelf. Estuarine Coastal and Shelf Science 84: 237–245.

[18] Rooper CN, Zimmermann M (2007) A bottom-up methodology for integrating underwater video and acoustic mapping for seafloor substrate classification. Continental Shelf Research 27: 947–957.

[19] Che Hasan R, Ierodiaconou D, Laurenson L (2012) Combining angular response classification and backscatter imagery segmentation for benthic biological habitat mapping. Estuarine Coastal and Shelf Science 97: 1–9.

[20] Ojeda GY, Gayes PT, Van Dolah RF, Schwab WC (2004) Spatially quantitative seafloor habitat mapping: example from the northern South Carolina inner continental shelf. Estuarine Coastal and Shelf Science 59: 399–416.

[21] Marsh I, Brown C (2009) Neural network classification of multibeam backscatter and bathymetry data from Stanton Bank (Area IV). Applied Acoustics 70: 1269–1276.

[22] Simons DG, Snellen M (2009) A Bayesian approach to seafloor classification using multi-beam echo-sounder backscatter data. Applied Acoustics 70: 1258–1268. 10.1121/1.3205397 19813788

[23] Folk RL (1954) The distinction between grain size and mineral composition in sedimentary-rock nomenclature. Journal of Geology 62: 344–359.

[24] Wilson MFJ, O'Connell B, Brown C, Guinan JC, Grehan AJ (2007) Multiscale Terrain Analysis of Multibeam Bathymetry Data for Habitat Mapping on the Continental Slope. Marine Geodesy 30: 3–35.

[25] Holmes KW, Van Niel KP, Radford B, Kendrick GA, Grove SL (2008) Modelling distribution of marine benthos from hydroacoustics and underwater video. Continental Shelf Research 28: 1800–1810.

[26] Venables W, Ripley B (2002) Modern Applied Statisitcs with S-PLUS. New York: Springer. 495 p.

[27] R Development Core Team (2011) R: A Language and Environment for Statistical Computing. R Foundation for Statistical Computing. 1706 p.

[28] Luts J, Ojeda F, Plas R, Van De Moor B, De Huffel S, et al (2010) A tutorial on support vector machine-based methods for classification problems in chemometrics. Analytica Chimica Acta 665: 129–145. 20417323 10.1016/j.aca.2010.03.030

[29] Meyer D (2012) Support Vector Machines: The Interface to libsvm pacakge e1071. Technische Universität Wien, Austria. 8 p.

[30] Therneau T, Atkinson E (1997) An Introduction to Recursive Partitioning Using the rpart Routine. Rochester: Section of Biostatistics, Mayo Clinic. 52 p.

[31] Breiman L (2001) Random Forests. Machine Learning 45: 5–32.

[32] Liaw A, Wiener M (2002) Classification and regression by random forest. R News 2/3: 18–22.

[33] Hastie T, Tibshirani R, Friedman J (2009) The Elements of Statistical Learning: Data Mining, Inference, and Prediction. New York: Springer. 745 p.

[34] John G, Langley P (1995) Estimating continuous distributions in Bayesian classifier. San Mateo, CA: Morgan Kaufmann. 338–345 p.

[35] Kursa M, Rudnicki W (2010) Feature selection with the Boruta Package. Journal of Statistical Software 36: 1–11.

[36] Kohavi R, Sommer D (1995) Subset Selection Using the Wrapper Method: Overfitting and Dynamic Search Space Topology Heuristic Search. Montreal: Canada. 192–197 p.

[37] Cohen J (1960) A Coefficient of Agreement for Nominal Scales. Educational and Psychological Measurement 20: 37–46.

[38] McKenzie DP, Mackinnon AJ, Peladeau N, Onghena P, Bruce PC, et al (1996) Comparing correlated kappas by resampling: Is one level of agreement significantly different from another? Journal of Psychiatric Research 30: 483–492. 9023792 10.1016/s0022-3956(96)00033-7

[39] Foody GM (2004) Thematic Map Comparison: Evaluating the Statistical Significance of Differences in Classification Accuracy. Photogrammetric Engineering & Remote Sensing 70: 627–633.

[40] Lüdtke A, Jerosch K, Herzog O, Schlüter M (2012) Development of a machine learning technique for automatic analysis of seafloor image data: Case example, Pogonophora coverage at mud volcanoes. Computers & Geosciences 39: 120–128.

[41] Li J, Heap AD, Potter A, Huang Z, Daniell JJ (2011) Can we improve the spatial predictions of seabed sediments? A case study of spatial interpolation of mud content across the southwest Australian margin. Continental Shelf Research 31: 1365–1376.

[42] Kohavi R, John GH (1997) Wrappers for feature subset selection. Artificial Intelligence 97: 273–324.

[43] Guyon I, Elisseeff A (2003) An Introduction to Variable and Feature Selection. Journal ofMachine Learning Research 3: 1157–1182.

[44] Harrison R, Birchall R, Mann D, Wang W (2012) Novel consensus approaches to the reliable ranking of features for seabed imagery classification. International Journal of Neural Systems 22: 1250026. 23186275 10.1142/S0129065712500268

[45] Collier JS, Brown CJ (2005) Correlation of sidescan backscatter with grain size distribution of surficial seabed sediments. Marine Geology 214: 431–449.

[46] Fonseca L, Brown C, Calder B, Mayer L, Rzhanov Y (2009) Angular range analysis of acoustic themes from Stanton Banks Ireland: A link between visual interpretation and multibeam echosounder angular signatures. Applied Acoustics 70: 1298–1304.

[47] Isaaks E, Srivastava R (1989) An introduction to applied geostatisitics. New York, Oxford: Oxford University Press. 561 p.

[48] Pingree RD, Griffiths DK (1979) Sand transport paths around the British Isles resulting from M_2_ and M_4_ tidal interactions. Journal of the Marine Biological Association of the United Kingdom 59: 497–513.

[49] Diesing M, Kubicki A, Winter C, Schwarzer K (2006) Decadal scale stability of sorted bedforms, German Bight, southeastern North Sea. Continental Shelf Research 26: 902–916.

[50] Kubicki A, Diesing M (2006) Can old analogue sidescan sonar data still be useful? An example of a sonograph mosaic geo-coded by the DECCA navigation system. Continental Shelf Research 26: 1858–1867.

[51] Last D (1992) The Accuracy and Coverage of Loran-C and of the Decca Navigator System - and the Fallacy of Fixed Errors. The Journal of Navigation 45: 36–51.

[52] Foody GM, Boyd DS, Sanchez-Hernandez C (2007) Mapping a specific class with an ensemble of classifiers. International Journal of Remote Sensing 28: 1733–1746.

[53] Lundblad ER, Wright DJ, Miller J, Larkin EM, Rinehart R, et al (2006) A Benthic Terrain Classification Scheme for American Samoa. Marine Geodesy 29: 89–111.

[54] Moran P (1950) Notes on continuous stochastic phenomena. Biometrika 37: 17–23. 15420245

